# No dramatic age-related loss of hair cells and spiral ganglion neurons in Bcl-2 over-expression mice or Bax null mice

**DOI:** 10.1186/1750-1326-5-28

**Published:** 2010-07-16

**Authors:** Haiyan Shen, Jonathan I Matsui, Debin Lei, Lirong Han, Kevin K Ohlemiller, Jianxin Bao

**Affiliations:** 1 Model Animal Research Center, Nanjing University, Nanjing, 210061, China; 2 Department of Otolaryngology, Washington University School of Medicine, St. Louis, MO, 63110, USA

## Abstract

Age-related decline of neuronal function is associated with age-related structural changes. In the central nervous system, age-related decline of cognitive performance is thought to be caused by synaptic loss instead of neuronal loss. However, in the cochlea, age-related loss of hair cells and spiral ganglion neurons (SGNs) is consistently observed in a variety of species, including humans. Since age-related loss of these cells is a major contributing factor to presbycusis, it is important to study possible molecular mechanisms underlying this age-related cell death. Previous studies suggested that apoptotic pathways were involved in age-related loss of hair cells and SGNs. In the present study, we examined the role of Bcl-2 gene in age-related hearing loss. In one transgenic mouse line over-expressing human Bcl-2, there were no significant differences between transgenic mice and wild type littermate controls in their hearing thresholds during aging. Histological analysis of the hair cells and SGNs showed no significant conservation of these cells in transgenic animals compared to the wild type controls during aging. These data suggest that Bcl-2 overexpression has no significant effect on age-related loss of hair cells and SGNs. We also found no delay of age-related hearing loss in mice lacking Bax gene. These findings suggest that age-related hearing loss is not through an apoptotic pathway involving key members of Bcl-2 family.

## Background

Functional decline of the nervous system is a cardinal feature of aging, yet the cellular mechanisms underlying this decline are unknown. The current view holds that age-related changes of neuronal connections, rather than neuronal loss, contribute to this functional decline in the central nervous system [1-3]. However, in the cochlea, age-related loss of auditory neurons, such as hair cells and spiral ganglion neurons (SGNs), is a major cause of presbycusis [4]. Similarly, age-related loss of hair cells and SGNs is observed in the inbred C57BL/6J mouse, an established animal model for presbycusis [5-7]. Despite this knowledge, the exact mechanisms underlying age-related death of hair cells and SGNs are unknown [8-11].

In general, cells die by either passive or active processes [12]. Necrosis is a passive process characterized by swelling of the cell body and spillage of the intracellular contents. Apoptosis is an active form of cell death characterized by a shrunken cell body and masses of condensed DNA. Although significant advances have been made in the study of cell death, clearly distinguishing between these two forms of cell death *in vivo *during aging has proven difficult [13,14]. Some cells, especially neurons, may show most of the hallmarks of apoptosis, but fail to show key properties, such as DNA laddering or condensation during death [15,16]. In spite of these limitations, the current view stands that, in the absence of manifest necrotic stimuli, most of the cell death during aging occurs via apoptosis, both in the brain [17,18] and cochlea [19-23].

Active cell death requires synthesis of new proteins and a programmed biochemical cascade. This cascade has been elegantly revealed through studies in *Caenorhabditis elegans*. Genetic analysis in this worm has identified several key cell-death (CED) genes [24-27]. The mammalian counterpart of one such gene, CED-9, has been identified as Bcl-2. In the auditory system, over-expression of Bcl-2 in transgenic mice prevents apoptosis in afferent deprivation-induced neuronal death of the anteroventral cochlear nucleus and aminoglycoside-induced hair cell death [28,29]. In contrast, Bax is a proapoptotic member of the Bcl-2 family [30-32], and deletion of the Bax gene (Bax-/-) reduces the incidence of naturally occurring neuronal apoptosis during development [31].

Given the technical difficulty of identifying apoptotic cells *in vivo *during aging, we have utilized a transgenic mouse line overexpressing Bcl-2 to examine the role of Bcl-2 in age-related death of hair cells and SGNs. We hypothesized that over-expression of Bcl-2 in hair cells and SGNs would delay age-related hearing loss if normal age-related death of hair cells and SGNs occurs through the apoptotic pathway involving this key member of the Bcl-2 family. Surprisingly, we found no effect of Bcl-2 overexpression on age-related hearing loss. In addition, the deletion of Bax also had no effect on age-related hearing loss. Thus, these findings indicate that normal age-related auditory neuronal loss in the cochlea is not mediated by this typical apoptotic pathway.

## Results

### Over-expression of human Bcl-2 in the hair cells and spiral ganglion neurons

**Figure 1:**
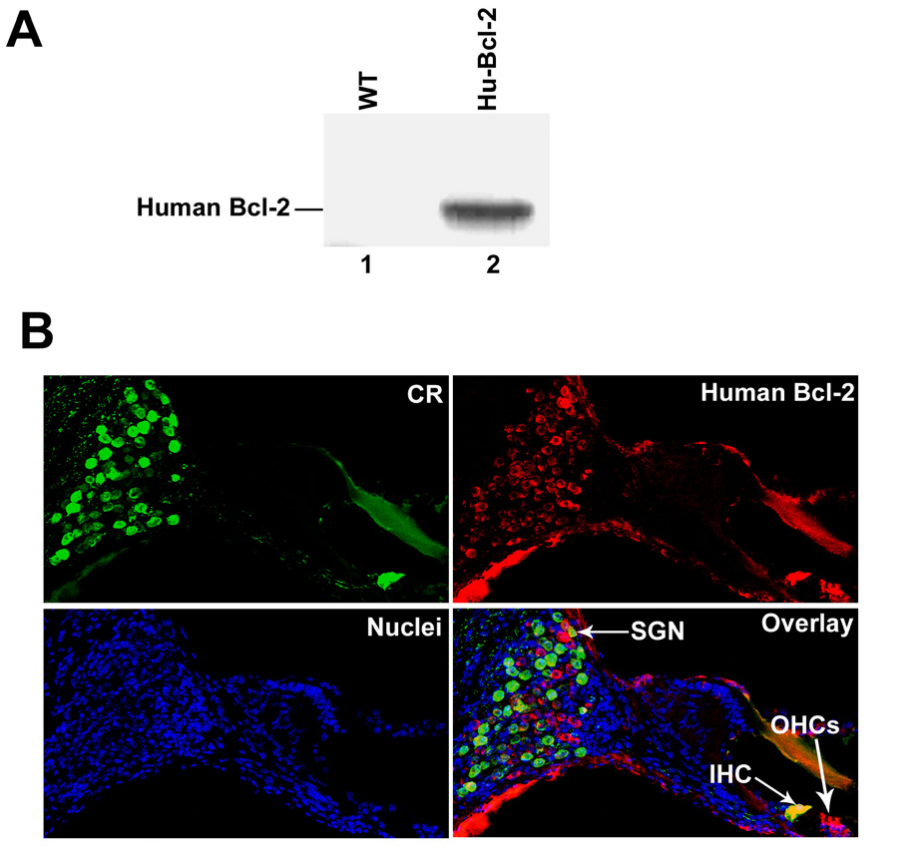
**Overexpression of human bcl-2 transgene in the mouse cochlea at four months old**. (A) Immunoblot analysis of human Bcl-2 expression. (1) A sample from two cochleae of C57BL/6J; (2) a sample from the transgenic mice. (B) Immunostaining of calretinin (CR) human bcl-2 in the cochlea of a transgenic mouse.

The transgenic mice used in this study express human Bcl-2 under the control of the neuron-specific enolase promoter [33]. We first examined the transgene expression of human Bcl-2 in mouse cochlea by Western blot with an antibody that recognizes only human Bcl-2 protein and does not cross-react with the mouse protein [34,35]. As expected, the human Bcl-2 was only detected in samples from transgenic mice (Figure 1A). To examine the expression pattern of the transgene in the cochlea, we further performed immunohistochemistry on cochleae from both wild-type and transgenic mice. Figure 1B shows a mouse cochlea that was double-labeled for calretinin, a marker for both inner hair cells and SGNs (green), and human Bcl-2 (red). Hair cells and SGNs from wild-type mice express calretinin but do not express human Bcl-2 (data not shown). Inner hairs cells and some SGNs from transgenic Bcl-2 overexpressing mice were immunoreactive for calretinin, and hair cells (including three outer hair cells) and SGNs from the same sample also expressed human Bcl-2 (Figure 1B). These data confirm that the transgenic mice overexpress human Bcl-2 in both hair cells and SGNs.

### Over-expression of Bcl-2 does not delay age-related hearing loss

**Figure 2:**
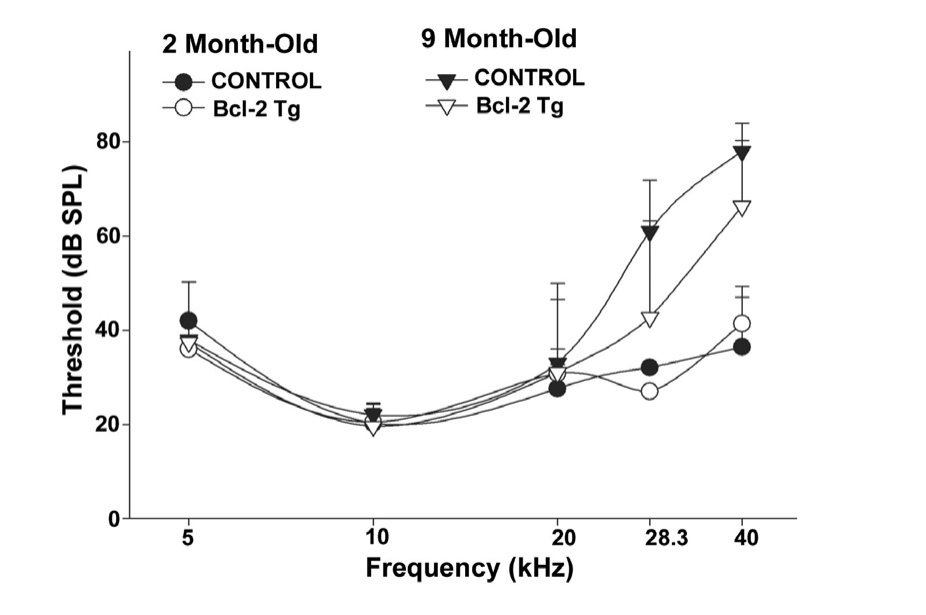
**Comparison of age-related hearing loss between wild-type and bcl-2 transgenic mice**. Mean (+SD) ABR thresholds were measured in wild type and BCL-2 transgenic mice versus age (2 and 9 months) and stimulus frequency. Five female mice were used for each group. No significant threshold protection effect of the BCL-2 transgene was apparent.

**Figure 3:**
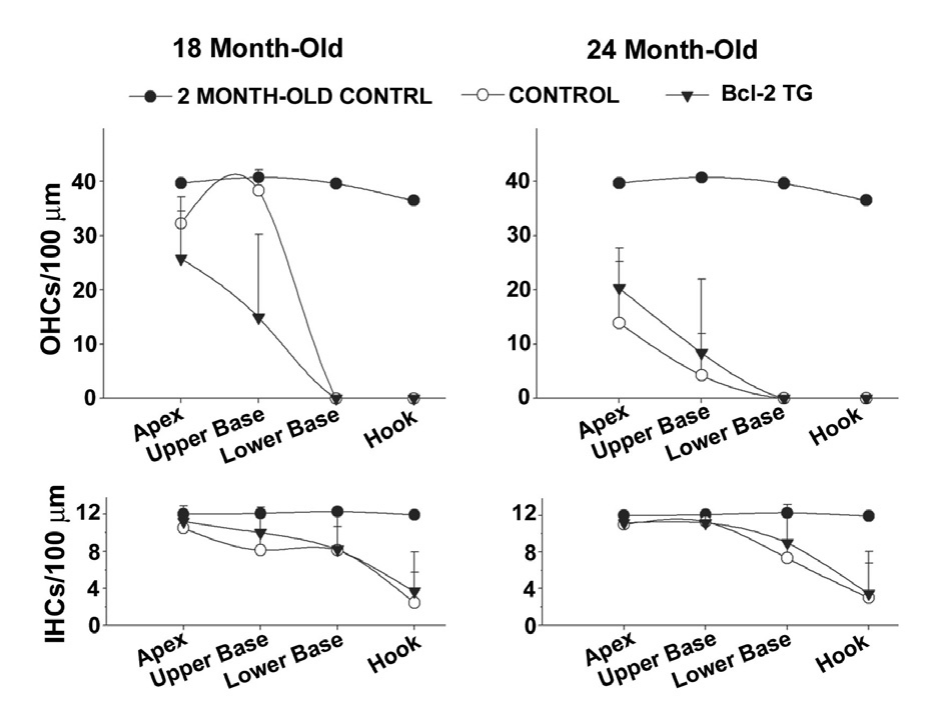
**Comparison of age-related loss of hair cells between wild-type and bcl-2 transgenic mice**. Mean (+SD) inner and outer hair cell density at four basal-apical locations versus age (18 and 24 months) and BCL-2 genotype. Averaged data for 2 month-old control female mice (n = 5), pooled by genotype, provided normal comparison numbers. Both wild type (n = 4) and transgenic mice (n = 4) showed progressive loss of inner (IHCs) and outer hair cells (OHCs), beginning in the base. There was no significant tendency for BCL-2 transgenic to retain hair cells.

To address whether overexpression of Bcl-2 in hair cells and SGNs during aging might delay age-related hearing loss, we compared ABR thresholds in wild type and transgenic mice at 2 and 9 months. Consistent with the C57 *Ahl *background [5], hearing thresholds increased with age, beginning with high frequencies. No significant effect of genotype was apparent (Figure 2A). To assess whether age-related loss of hair cells was delayed by over-expression of human Bcl-2, we examined the number of hair cells in both wild type and transgenic mice from 18- and 24-month-old mice. Figure 3 compares hair cell densities of wild type and transgenic mice at four basal-apical locations. Pooled counts from two-month-old animals provided normal densities for comparison. Both inner and outer hair cells progressively degenerated with age, following a basal-to-apical pattern. Counts obtained at 18 and 24 months revealed no indication that survival of either inner or outer hair cells was greater in transgenic mice. Trends suggested by the data, including better survival of OHCs in wild types at 18 months and better survival of OHCs in transgenic at 24 months, were not significant.

**Figure 4:**
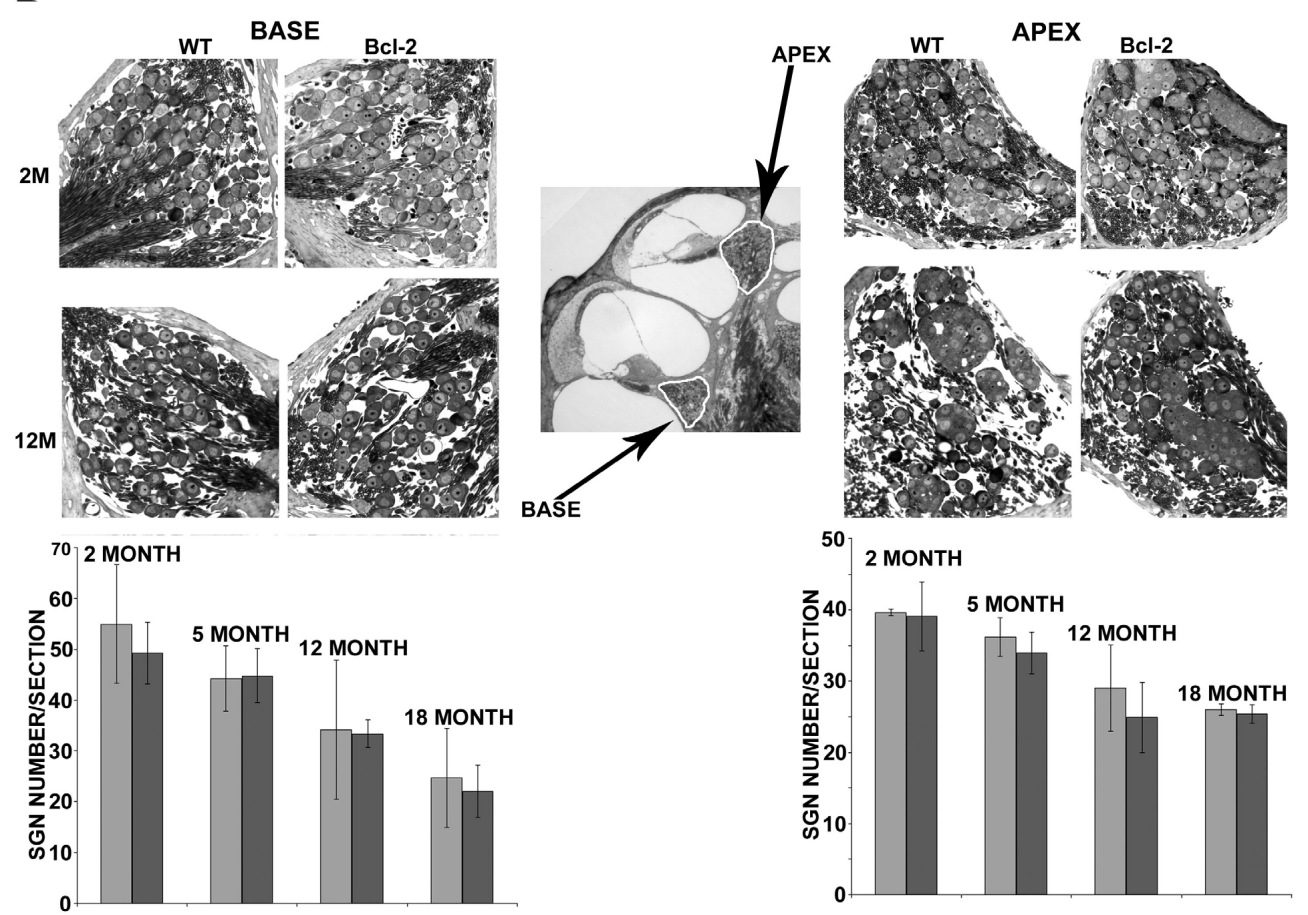
**Comparison of age-related loss of hair cells and SGNs between wild-type and bcl-2 transgenic mice**. Histological sections of spiral ganglia of wild-type (WT) and transgenic mice (Bcl-2) from 2 or 12 month-old age groups were shown. There were four animals in each group. The spiral ganglia at the base are shown in the left panel, and the ganglia in the apex at the right panel. SGNs were counted from each age group and presented below the histological sections.

To assess whether age-related loss of SGNs was delayed by over-expression of human Bcl-2, we compared the number of SGNs between wild type and transgenic mice at 2, 5, 12, and 18 months old. In general, SGN loss increased with age. Similar to the observation from hair cells, no difference was observed between wild type and transgenic mice for age-related loss of SGNs (Figure 4). At the base of the cochlea, there was no difference in the number of SGNs and their general morphological features between wild type and transgenic mice from two to eighteen months old (Figure 4, left panel). A similar observation was made at the apex of the cochlea (Figure 4, right panel). The rate of age-related loss of SGNs at the base of the cochlea was higher than that of the apex.

### No difference in hearing loss between control and Bax-/- mice

**Figure 5:**
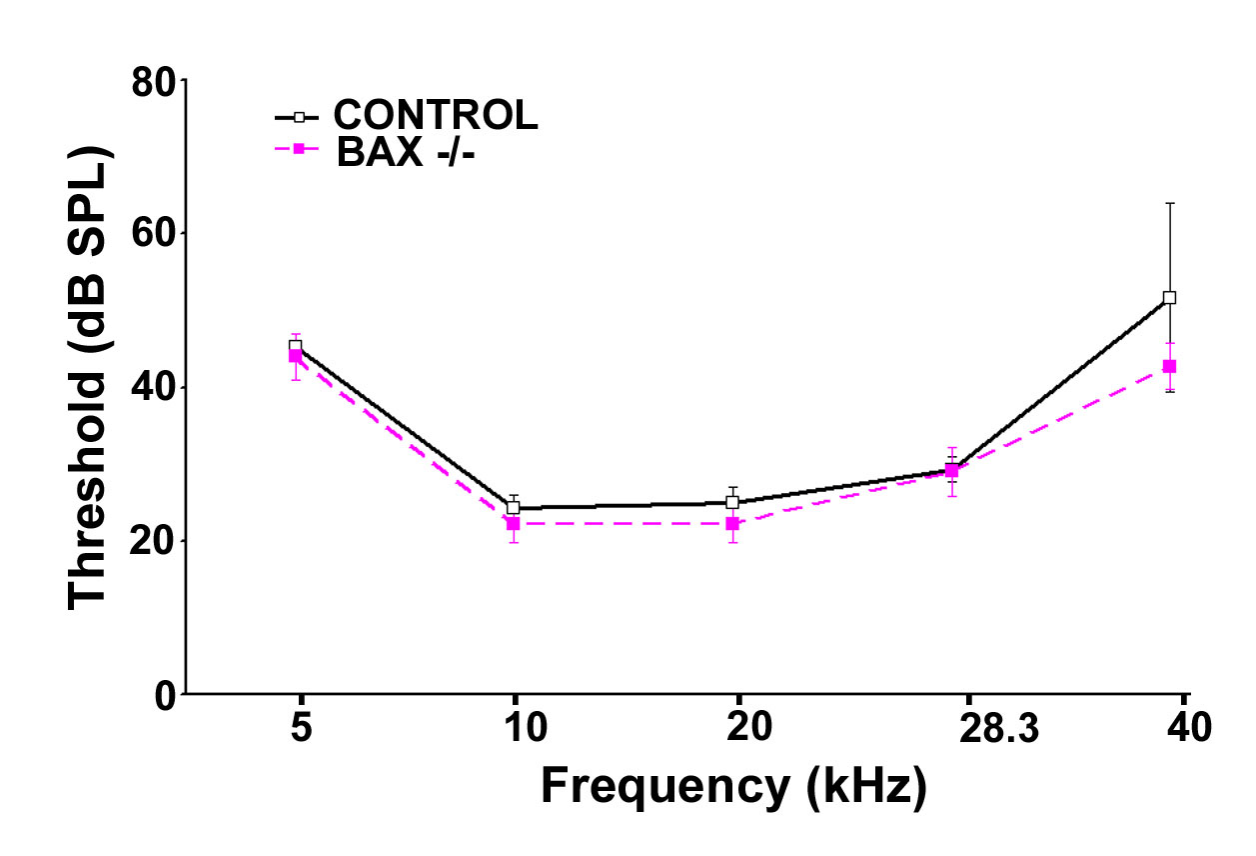
**Comparison of age-related hearing loss between wild-type and Bax-/- mice**. Mean (+SD) ABR thresholds were measured in both wild type (n = 3) and Bax-/- mice (n = 4). They were 5 month-old male mice. No significant threshold protection effect of the BCL-2 transgene was apparent.

**Figure 6:**
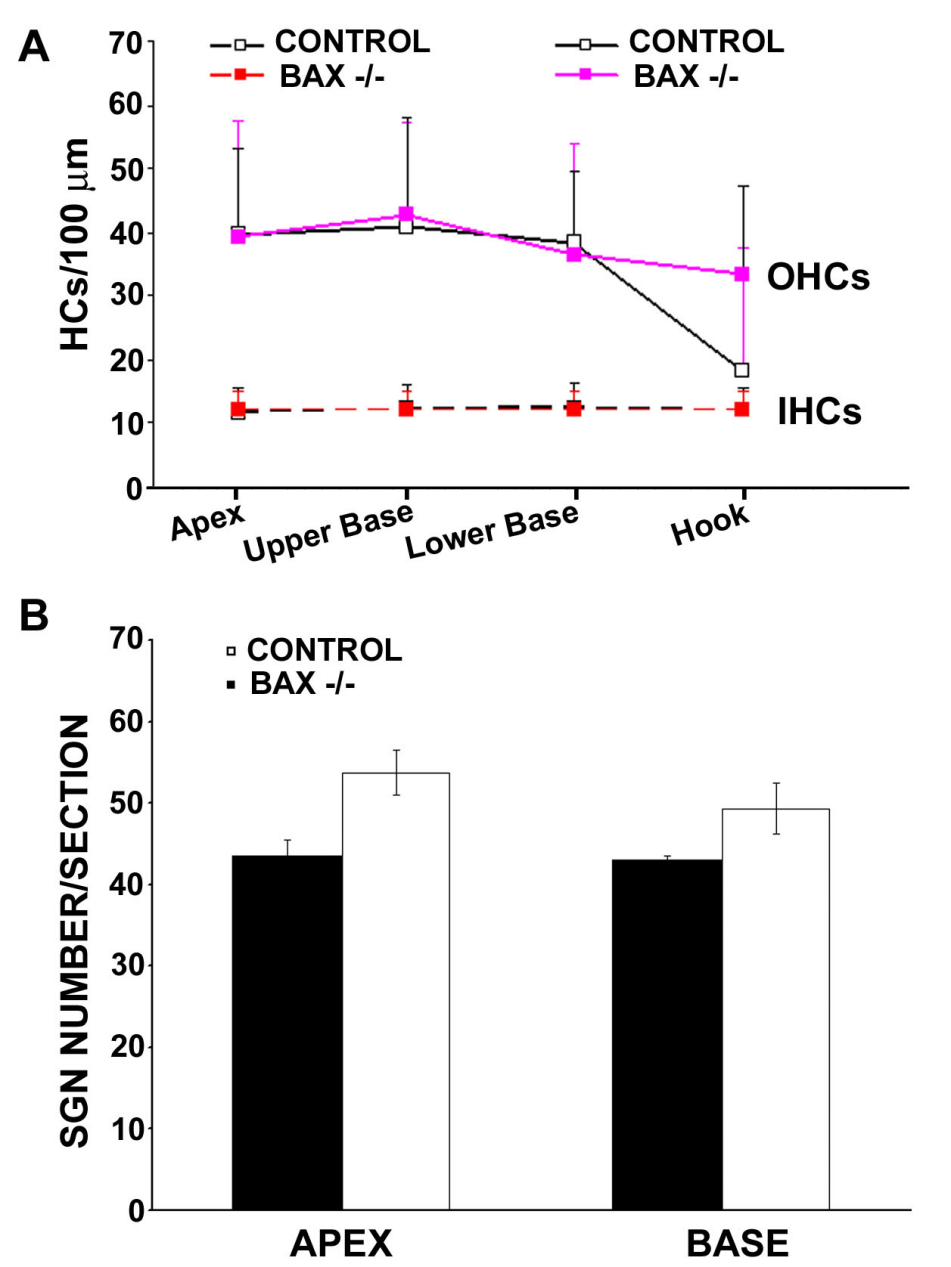
**Comparison of age-related loss of hair cells and SGNs between wild-type and Bax-/- mice**. (A) Mean (+SD) inner and outer hair cell density at four basal-apical locations from the same mice tested in Figure 5. (B) Comparison of SGN numbers between wild-type and Bax-/- mice. The number of SGNs were counted and compared at both the apex and base region from the same 5 month-old mice.

No delay of age-related loss of hair cells and SGNs was also found in Bax-/- mice [22], and Bax is another key member of Bcl-2 family. We also determined whether there was no delay of age-related hearing loss in Bax-/- mice with assessment of hearing threshold by ABR to tone stimuli at 5, 10, 20, 28.3, and 40 kHz. As expected, there were no significant differences in ABR thresholds between Bax-/- and control mice at five months old (Figure 5). Furthermore, there were no significant differences in the number of inner hair cells (IHCs), outer hair cells (OHCs), and SGNs between the control and Bax-/- mice (Figure 6A and 6B). We noted that there tended to be more OHCs at the hook region for the Bax null mice, however, this difference was not statistically significant. Most interestingly, there tended to be more SGNs in the control mice instead. Thus, consistent with the previous study [22], Bax targeted deletion did not delay age-related loss of hair cells and SGNs.

## Discussion

Our results demonstrate that neither the deletion of Bax gene nor over-expression of Bcl-2 in hair cells and SGNs delays age-related hearing loss. Despite Bax-/- or overexpression of Bcl-2 in hair cells and SGNs, age-related death of these cells still occurred at a rate similar to the cells in wild-type controls. Thus, our data challenges the position that the apoptosis pathway involving the Bac-2 family is the molecular mechanism underlying normal age-related neuronal loss.

Since it is still uncertain whether there is age-related neuronal loss in the central nervous system, it is difficult to interpret changing expression levels of various apoptotic genes in the brain during aging [17,36]. However, in the cochlea, age-related loss of hair cells and SGNs are consistently observed across species [5,6,8]. Several studies have suggested a role for apoptosis in age-related loss of hair cells and SGNs. Using the terminal deoxynucleotidyl transferase-mediated dUTP-biotin nick end-labeling (TUNEL) method, previous studies found the presence of DNA fragmentation in the hair cells and SGNs during aging [37,38]. However, DNA fragmentation could also be caused by necrosis. Further evidence to support age-related loss of hair cells and SGNs through apoptosis came from an association of aging with the expression of apoptosis-related proteins in the cochlea [19,20]. It was unclear whether changes in the expression of apoptosis-related proteins were due to the expression changes or loss of auditory neurons during aging. Another indirect piece of evidence was a significant reduction in the number of TUNEL-positive cells and cleaved caspase-3-positive cells in the cochlea from calorie restriction mice [21].

Thus, the central question of whether age-related death of hair cells and SGNs occurs via apoptosis has persisted. In Bax-/- mice or mice overexpressing Bcl-2, we found no difference in age-related hearing loss compared to their genetic background matched control. Furthermore, earlier work showed that loss of hair cells and SGNs in one-month-old mice lacking caspase-3, a key downstream caspase in the apoptotic cascade, suggesting that death of hair cells and SGNs does not need caspase-3 [39]. Considering all these findings, our data provides insight into the question of whether normal age-related loss of auditory neurons is due to the apoptosis pathway involving members of Bcl-2 family.

During early development, 20-80% of neurons die through apoptosis, which appears to be due to competition for trophic factors from target tissues [40]. Early neuronal death can be prevented by the overexpression of anti-apoptotic protein Bcl-2 or by the deletion of pro-apoptotic proteins Bax or caspase 3 [33,41,42]. During aging, the target of SGNs, hair cells, die first, followed by withdrawal of the afferent fibers of SGNs, leading to the lack of trophic supports for SGNs [6]. Thus, it is intriguing that no effect of Bax-/- or Bcl-2 over-expression was found on age-related loss of SGNs, assuming they die through apoptosis. Since there are other anti-apoptosis pathways such as oxidative stress induced pathways [22] and "extrinsic" pathways induced by Fas/FasL [23,43], we cannot conclusively exclude other apoptosis pathways as the cause of age-related loss of hair cells and SGNs. Our studies suggest that it is worth also to explore possible involvements of other two types of cell death, autophagy and necrosis, in age-related neuronal loss.

## Conclusion

In summary, our results show that, in mice, age-related loss of auditory neurons -hair cells and SGNs - cannot be delayed by Bax deletion or over-expression of Bcl-2. Despite extensive research on the functional decline of the nervous system, there is conflicting evidence regarding whether age-related neuronal loss occurs through a typical apoptotic pathway. The data from this study, in combination with other genetic studies, suggest that age-related loss of hair cells and SGNs is not dependent on the functions of several key proteins in the apoptotic pathway, such as Bcl-2, Bax, and caspase-3. However, alternative apoptotic pathways may contribute to age-related neuronal loss. Further understanding of the possible roles for alternative apoptotic pathways or other forms of cell death in age-related loss of hair cells and SGNs will help elucidate the processes underlying age-related neuronal loss and provide new strategies for delaying functional decline of the nervous system.

## Methods

### Animals

Mice lacking Bax and mice expressing the human Bcl-2 gene under the control of the neuron-specific enolase (NSE) promoter were bred in the CID animal care facility. All experimental protocols were approved by the appropriate Institutional Animal Care and Use Committee (Washington University/CID). In order to reduce the possibility of effects of other genes co-segregating with the bcl-2 transgene, Bax-/- and Bcl-2 overexpressing transgenic mice were backcrossed to C57BL/6J for at least 8 generations prior to the onset of our studies. The genotype of each mouse was determined by tail-clip DNA analysis using PCR.

### Auditory Brainstem Recording (ABR) Testing

ABR testing was performed in a foam-lined, double-walled soundproof room (Industrial Acoustics). Animals were anesthetized (80 mg/kg ketamine, 15 mg/kg xylazine, i.p.) and positioned dorsally in a custom head holder. Core temperature was maintained at 37°C using a thermostatically controlled heating pad in conjunction with a rectal probe (Yellow Springs Instruments Model 73A). Platinum needle electrodes (Grass) were inserted subcutaneously just behind the right ear (active), at the vertex (reference), and in the back (ground). Electrodes were led to a Grass P15 differential amplifier (100 Hz-10 kHz, X100), then to a custom broadband amplifier (0.1 Hz-10 kHz, X1000), and digitized at 30 kHz using a Cambridge Electronic Design micro1401 in conjunction with SIGNAL and custom signal averaging software operating on a 120 MHz Pentium PC. Sinwave stimuli generated by a Wavetek Model 148 oscillator were shaped by a custom electronic switch to 5 ms total duration, including 1 ms/rise/fall times. The stimulus was amplified by a Crown D150A power amplifier and output to a KSN1020A piezo ceramic speaker located 7 cm directly lateral to the right ear. Stimuli were presented freefield and calibrated using a 1/4 inch microphone (B&K 4135) placed where the pinna would normally be. Toneburst stimuli at each frequency and level were presented 1000 times at 20/s. The minimum sound pressure level required for a response (short-latency negative wave) was determined at 5, 10, 20, 28.3 and 40 kHz, using a 5 dB minimum step size.

### Tissue Preparation

Mice were transcardially perfused with 2% paraformaldehyde and 2% glutaraldehyde in 0.1 M sodium phosphate, PH 7.6. The cochlea was post-fixed overnight in the same solution and decalcified in sodium EDTA. For immunocytochemistry, cochlea samples were immersed in OCT compound (Sakura Finetek USA, Torrance, CA) and frozen on dry ice. 10 μm cryostat sections were cut and immunostained with monoclonal hamster anti-human Bcl-2 antisera (1:100, PharMingen). For general histology, cochlea samples were post-fixed in buffered 1% osmium tetroxide, dehydrated in an ascending acetone series, and embedded in Epon.

### Hair Cell Counts

Twenty right cochleae from wild type and transgenic mice were dissected into roughly half-turn segments. Segments were immersed in mineral oil and viewed as surface preparations using Nomarski optics and a calibrated ocular. Separate IHC and OHC counts were obtained at each of four locations: apical (.5-1.0 mm from the apex); upper base (2.0-3.0 mm from the apex); lower base (3.5-4.5 mm from the apex); and hook region (most basal 1.0 mm). Hair cells were counted in three 100 μm segments. Counts were averaged across animals by genotype and age. In two-month old mice, inner and outer hair cell densities were within normal limits, based on previous work, independent of genotype. Data from these animals were therefore pooled to provide a 'normal' standard.

### SGN Counts

For SGN counting, left cochleae were cut parallel to the mid-midiolar plane at 4.0 μm. All nuclei were counted from the spiral ganglia located at basal or apex regions on every 4^th ^section, similar to previously published methods [5]. Significant differences of SGN numbers between wild-type and transgenic mice were analyzed by t-test.

## Competing interests

The authors declare that they have no competing interests.

## Authors' contributions

HS, JIM, DL, KKO and JB designed the experiments, statistical analysis, interpreted the results and drafted the manuscript. HS LH, and DL carried out the experiments. KKO and JB drafted the manuscript. All authors read and approved the final manuscript.
